# Do Tobacco Treatment Trials Address Disparities in Smoking Outcomes Among Black and Hispanic Cancer Patients? A Systematic Review of Smoking Cessation Interventions for Black and Hispanic Patients Diagnosed with Cancer

**DOI:** 10.1007/s40615-023-01705-3

**Published:** 2023-07-19

**Authors:** Giselle K. Perez, Julia T. Rabin, Megha Tandon, Nicole M. Strauss, Kelly Irwin, Lisa Philpotts, Jamie Ostroff, Elyse R. Park

**Affiliations:** 1 Harvard Medical School/Massachusetts General Hospital, Boston, MA USA; 2 Health Promotion and Resilience Intervention Research Program, 100 Cambridge Street, 16th floor, Boston, MA 02114 USA; 3 Department of Psychology, University of Cincinnati, Cincinnati, OH USA; 4 Tufts University, Medford, MA USA; 5 Bamboo Health, Boston, MA USA; 6 Memorial Sloan Kettering Cancer Center, New York, NY USA

**Keywords:** Tobacco treatment, Tobacco and health, Cancer disparities, Clinical trial participation

## Abstract

**Objective:**

To characterize the representation of Black and Hispanic cancer patients in tobacco treatment trials, and to offer recommendations for future research.

**Methods:**

We conducted two systematic searches of the literature (2018, 2021) using 5 databases (MEDLINE via EBSCO, Pubmed, PsycInfo, Cumulative Index to Nursing and Allied Health Literature (CINAHL), Excerpta Medica Database (EMBASE)) to examine the prevalence of tobacco trials that included Black or Hispanic cancer patients. Two coders independently screened all articles at title, abstract, and full-text to identify eligible trials. Information about the proportion of Black and Hispanic patients included, trial design features, and whether the authors analyzed outcomes for Black and Hispanic patients were documented.

**Results:**

Of 4682 identified studies, only 10 published trials included and reported on the rates of Black or Hispanic cancer patients enrolled in their tobacco trial. The proportion of enrolled Black cancer patients ranged from 2 to 55.6%. Only our studies documented enrollment rates for Hispanics, and rates were less than 6%. None of the studies offered strategies to promote or the accrual of Black or Hispanic patients.

**Discussion:**

There remains a large gap in the literature regarding the reach and efficacy of tobacco treatment for Black and Hispanic cancer patients. Black and Hispanic cancer patients remain largely under-represented in tobacco cessation trials, limiting the applicability of existing, evidence-based treatments. To optimize intervention generalizability, future studies should emphasize the targeted recruitment and engagement of these patients in tobacco trials.

**Supplementary Information:**

The online version contains supplementary material available at 10.1007/s40615-023-01705-3.

## Introduction

Blacks and Hispanics living in the USA face increased risk for cancer and for worse cancer outcomes, including worse survival [1–3]. Cigarette smoking, albeit modifiable, is a known risk factor for at least 12 types of cancers. Despite demonstrating greater interest and efforts to quit, Black and Hispanic smokers are less likely to receive quit advice; initiate, participate in, and comply with tobacco treatment; utilize pharmacological agents to aid cessation efforts; and maintain abstinence after quitting [4–8]. For smokers diagnosed with cancer, continued smoking has the potential to aggravate cancer care, leading to treatment-related side effects, poorer quality of life, increased risk of recurrence, and adverse cancer outcomes [9–12]. Although 10–30% of cancer patients report continued smoking after a cancer diagnosis [13, 14], given the prevailing evidence, it is possible that these rates are disproportionately higher among Black and Hispanic cancer patients.

A number of researchers have endeavored to decrease the burden of cancer by tailoring tobacco trials to the needs of cancer patients who smoke; yet, it remains unknown if these interventions translate to improvements in smoking outcomes among these two vulnerable groups. This is a critical question; despite national mandates (including the 1993 NIH Revitalization Act), ethnic and racial minorities are generally under-represented in cancer clinical trials, challenging the accuracy and generalizability of trial findings [15]. If issues of non-inclusion or under-representation of Black and/or Hispanic cancer patients extend to tobacco treatment trials, it may risk widening disparities in cancer outcomes [16, 17]. In response to this concern, this systematic review searched and synthesized the literature to (1) characterize the general reporting of Black and Hispanic cancer patients enrolled in tobacco treatment trials, (2) document the proportion of Black and Hispanic patients enrolled, (3) examine the “fit” between a trial’s design and the unique needs of Black and Hispanic cancer patients who smoke, and (4) summarize the reporting of tobacco treatment effects for Black and Hispanic cancer patients. Specifically, this review seeks to answer key questions surrounding the existing tobacco treatment evidence base for tobacco-dependent Black and Hispanic cancer patients, such as: Do existing tobacco treatment trials *include* Black and Hispanic cancer patients? Are tobacco treatment trials *accessible* to this population (i.e., are Black and Hispanic patients eligible and able to participate; are trials offered in community settings where these patients commonly present for care; are culturally sensitive study materials and multilingual staff used to promote engagement?)? To what extent do investigators document the use of culturally sensitive approaches to ensure the inclusion and engagement of Black and Hispanic cancer patients? To what extent do investigators address racial/ethnic variables that influence patients’ willingness to participate? Do trials demonstrate equitable efficacy in improving cessation rates in these groups? Understanding the successes and challenges of providing tobacco support to Black and Hispanic cancer patients in the cancer context will help guide the development of efficacious, culturally sensitive smoking cessation interventions that comprehensively address the complex needs of these two vulnerable groups.

## Methods

This systematic review was conducted in accordance with the Preferred Reporting Items for Systematic Review and Meta-Analysis (PRISMA) Guidelines from 2009. A PRISMA protocol was created and registered under the PROSPERO database (registration number CRD42016050547).

### Literature Search Strategy

Searches were conducted by a reference librarian (LP) in the following databases with publication dates ranging from the date of inception to January 2021: MEDLINE via EBSCO, Pubmed, PsycInfo, CINAHL, and Embase. Both controlled vocabulary and text word searches were conducted, as appropriate. To identify and maximize study eligibility, search terms included, but were not limited to the following terms: Hispanic, African American, Black, cessation, intervention, smoking, tobacco, cigarette, and cancer. Members of the study team compiled lists of search terms specific for each database. A qualified research librarian completed a preliminary search of all available articles published on the topic through 05/02/2018 and a second search through (01/15/21), only including studies written in English. A complete description of our Medical Subject Headings (MeSH) and keyword terms, as well as our exact search strategy, is available upon request. Two independent researchers (JR, NS) also examined the bibliographies of included studies to ensure no additional trials met the inclusion criteria.

### Eligibility

**Fig. 1:**
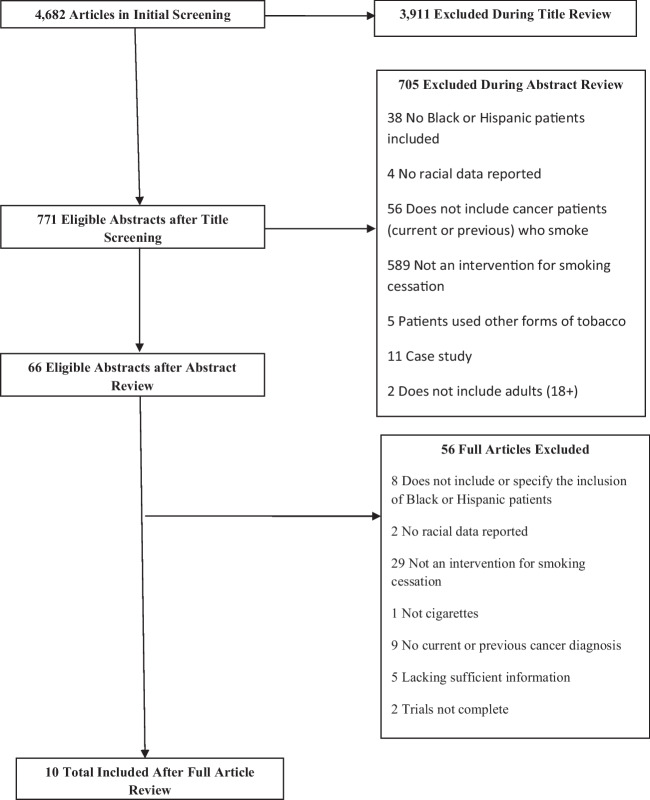
PRISMA Flow Diagram

Any smoking cessation intervention trial was eligible for inclusion if participants were adults, had a current or previous cancer diagnosis, and were current cigarette smokers. We excluded case studies as well as trials that did not specify the inclusion of Black or Hispanic participants in their sample. Trials that collapsed all racial and ethnic categories when reporting the demographic make-up of their sample were excluded. Studies were also excluded if participants only smoked other forms of tobacco and not cigarette use. We additionally excluded studies conducted outside of the USA due to differences in racial/sociodemographic make-up and challenges across countries (Fig. 1 outlines eligibility/exclusion breakdown). We did not use time frame restrictions in this study.

### Search Strategy and Extraction

To begin the multi-step process to determine eligibility, two independent researchers (BT and NS) conducted an initial screening of all available articles at the title, abstract, and full-text levels. Two additional researchers (JR and NS) completed a secondary review to resolve remaining discrepancies at each of the three levels of screening. These same researchers completed an additional review of all emerging articles since May 2018 through January 2021 to ensure that all eligible articles were included in this systematic review. Articles were discussed in detail among the study team if questions regarding eligibility arose. Figure 1 details this three-tiered process.

After the exclusion and inclusion process, the study team extracted data from eligible studies (*n* = 10) related to the enrollment and engagement of Black and Hispanic cancer patients in tobacco treatment trials. We created data spreadsheets to organize and extract relevant information from each article. Specifically, our study team extracted data related to the proportion of Black and Hispanic cancer patients enrolled, the type and location of the study, screening and recruitment strategies, identification of culturally sensitive intervention approaches, and reporting of racial/ethnic sub-analyses. In studies that reported a catchment area, census data from the relevant decade was used to ascertain the racial/ethnic demography of the catchment area and compare it to the participant demographics (Table 1). Due to the limited number of eligible articles and the heterogeneity of the study population and treatment designs, findings are summarized narratively rather than statistically.

**Table 1:** Summary of studies included in the systematic review of smoking cessations interventions for Black and Hispanic cancer patients

Author year	Article title	Study type	Single or multi-site	Setting (hospital vs. community)	Sample size	Race *n*, (%)	Catchment area (%)
Browning et al., 2000	Implementing the Agency for Health Care Policy and Research's Smoking Cessation Guideline in a Lung Cancer Surgery Clinic	Quasi-experimental design	Multi-site	Hospital (Arthur G. James Cancer Hospital, Solove Research Institute)	Total *n* = 25 (intervention group = 14; usual care group = 11)	White = 21 (84) Black = 4 (16) Hispanic = n/a	Columbus, OH^18^ White = 483,332 (67.9) Black = 174,065 (24.5) Hispanic = 17,471 (2.5)
Nair et al. 2009	Smoking Cessation Among Patients in a Cancer Clinic: Evaluation of a Novel, Motivational Stop-Smoking Pocket Calendar	Prospective cohort	Single	Community (Central Arkansas Veterans Healthcare System)	Total n = 32 (intervention = 32)	White = 27 (85) Black = 5 (15) Hispanic = n/a	Central Arkansas, AR^18^ White = 434,848 (74.5) Black = 127,737 (21.9) Hispanic = 12,337 (2.1)
Schnoll et al., 2010	A Bupropion Smoking Cessation Clinical Trial for Cancer Patients	RCT	N/A	Hospital	Total n = 246 (depression symptom placebo = 27; non-depression symptom placebo = 105; depression symptom bupropion = 28; non-depression symptom bupropion = 86)	White = 162 (66) Black = 71 (29) Other = ~ 13 (5) Hispanic = n/a	Unknown
Schnoll et al., 2003	Brief Physician-Initiated Quit-Smoking Strategies for Clinical Oncology Settings: A Trial Coordinated by the Eastern Cooperative Oncology Group	RCT	Multi-site	Community (Eastern Cooperative Oncology Group, Southwestern Oncology Group, Cancer and Leukemia Group B)	Total *n* = 432 (usual care = 217; physician-based intervention = 215)	White = 313 (73) Black = 10 (2) Native American = 3 (≤ 1) other/unknown = 105 (24.3)Hispanic = 1 (≤ 1)	Philadelphia, PA^18^ White = 683,267 (45.0) Black = 655,824 (43.2) Hispanic = 128,928 (8.5)
Gritz et al., 1993	Predictors of Long-Term Smoking Cessation in Head and Neck Cancer Patients	RCT	Multi-site	Hospital (UCLA and other)	Total *n* = 186 (completers = 114; non-completers = 72)	White = 135 (73) Black = 35 (19) Other = 16 (9) Hispanic = n/a	Los Angeles and Ventura County, CA^19^ White = 5,564,269 (58.4) Black = 1,008,243 (10.6) Hispanic = 3,528,194 (37.0)
Schnoll et al., 2005	A Randomized Pilot Study of CBT Versus Basic Health Education for Smoking Cessation Among Cancer Patients	RCT	N/A	Hospital	Total n = 109 (cognitive behavioral therapy = 52; general health education = 57)	White = 98 (90) Black = 10 (9) Hispanic = 1 (≤ 1)	Unknown
Park et al., 2020	Effect of Sustained Smoking Cessation Counseling and Provision of Medication vs Shorter-term Counseling and Medication Advice on Smoking Abstinence in Patients Recently Diagnosed With Cancer: A Randomized Clinical Trial	RCT	Multi-site	Hospital (Massachusetts General Hospital/Dana-Farber/Harvard Cancer Center and Memorial Sloan Kettering Cancer Center)	Total *n* = 303 (intensive treatment *n* = 153; standard treatment *n* = 150)	American Indian = 3 (0.9) Asian = 2 (0.7) Black, non-Hispanic = 20 (6.6) Black, Hispanic = 1 (0.3) White, non-Hispanic = 255 (84.2) White, Hispanic = 10 (3.3) other = 2 (0.7)	Boston, MA^20^ White = 365,693 (52.8) Black = 174,535 (25.2) Hispanic = 137,135 (19.8) Asian = 67,182 (9.7) American Indian = 2078 (0.3) New York, NY White = 2,676,118.26 (32.1) Hispanic = 2,426,014 (29.1) Black = 2,025,847 (24.3) Asian = 1,175,491 (14.1) American Indian = 33,347 (0.4)
Ruebush et al., 2020	Using a Family Systems Approach to Treat Tobacco Use among Cancer Patients	Pilot	Single	Hospital (North Carolina Cancer Hospital)	Total *n* = 532 (family integrated = 221, family present, not integrated = 21, family not present = 290)	White = 368 (69.2) Black or African American = 121 (22.7) American Indian or Alaska Native = 8 (1.5) Asian = 2 (0.4) unknown = 33 (6.2)	Chapel Hill, NC^20^ White = 45,925 (71.7) Black = 6982 (10.9) American Indian = 192 (0.3) Asian = 8327 (13)
Charlot et al., 2019	Feasibility and Acceptability of Mindfulness-Based Group Visits for Smoking Cessation in Low-Socioeconomic Status and Minority Smokers with Cancer	Cohort	Single	Community (Boston Medical Center Outpatient Clinic)	Total *n* = 18 (cohort 1 *n* = 8, cohort 2 *n* = 10)	non-Hispanic White = 8 (44.4) non-Hispanic Black = 10 (55.6)	Boston, MA^20^ White = 365,693 (52.8) Black = 174,535 (25.2) Hispanic = 137,135 (19.8)
Krebs et al., 2018	The QuitIT Coping Skills Game for Promoting Tobacco Cessation Among Smokers Diagnosed With Cancer: Pilot Randomized Controlled Trial	RCT	Single site	Hospital (Memorial Sloan Kettering)	Total enrolled *n* = 38	White *n* = 35 (92.1) Black *n* = 1 (2.6) Hispanic *n* = 2 (5.2)	New York, NY^20^ White = 2,676,118.26 (32.1) Hispanic = 2,426,014 (29.1) Black = 2,025,847 (24.3)

### Quality Assessment

We used the NIH Quality Assessment of Controlled Intervention Studies and the NIH Quality Assessment Tool for Before-After (Pre-Post) Studies with No Control Group to determine the quality ratings of the eligible studies [18, 19]. Two researchers independently rated each article as good, fair, and/or poor based on the NIH Quality Assessment. This Assessment addressed treatment allocation, blinding group assignments, drop-out rate, adherence to protocols, outcome assessment and analysis, and other sources of bias. The team discussed rating discrepancies until agreement was reached for all studies.

## Results

### Prevalence of Tobacco Treatment Trials Including Black and Hispanic Cancer Patients

Approximately 4682 articles were identified, and 3911 were excluded after title review. Furthermore, 771 abstracts were assessed, of which 66 underwent further review. Of the 66 articles reviewed at full-text, Black or Hispanic cancer patients were represented in only 10 (15.2%) studies. Accordingly, only 10 articles met eligibility criteria and were included in this review (Fig. 1).

Quality Assessment. We determined that 2/10 (20%) studies were at high risk for bias (see Table 2). Factors that impacted the quality of the study included randomization, study type, blinding, drop-out rate, and the use of valid and reliable measures. Eight studies were considered to have low risk of bias [20–27].

**Table 2:** Outcomes and subgroup analyses focused on racial/ethnic minorities and drop-out rates of included studies

Author and year	Primary outcome	Secondary and/or exploratory outcomes	Subgroup analyses	Drop-out rate	Quality assessment
Browning et al., 2000	Self-report and biochemical confirmation six months post-surgery (CO) for intervention vs. control (usual care) were 71% and 55% respectively (*p* = 0.383)	*There were no secondary or exploratory outcomes in this study*	None	One patient died shortly after 6-month follow-up in the usual care condition. One patient passed away in the intervention group one month prior to the 6-month follow-up (who was counted as a smoker even though 5-month follow-up confirmed smoking abstinence)	Fair
Nair et al., 2009	24% (5 of 21) of patients had quit smoking at 3-month survey follow-up	Average quantity of cigarettes smoked decreased from 25.3 to 15.8 (*p* = 0.0001)	None	2 patients died before completing the second questionnaire	Poor
Schnoll et al., 2010	Treatment arm did not significantly influence 7-day point prevalence abstinence (using CO) at 12 weeks (24.2% placebo vs. 27.2 bupropion) or 27 weeks (17.4 placebo vs. 18.4% bupropion)	For patients with depression (score CESD > 16), those who received bupropion had decreased withdrawal symptoms (Minnesota nicotine withdrawal scale) (*p* = 0.002) and increased QOL (SF-12) (*p* = 0.001). Patients with depression reported lower abstinence rates as compared to those without depression (*p* = 0.03)	None	Placebo CESD score > 16 = 7 withdrew, 3 unreachable. Placebo CESD score < 16 = 10 withdrew, 17 unreachable bupropion CESD score > 16 = 4 withdrew, 5 unreachable buproprion CESD score < 16 = 13 withdrew, 18 unreachable *percentage of total *n*	Good
Schnoll et al., 2003	At 6 months, 14.4% vs. 11.9% of participants in the intervention vs. usual care reported 7-day point prevalence abstinence At 12 months, 13.3% vs. 13.6% of participants in the intervention vs. usual care reported 7-day point prevalence abstinence	Above 16 years of smoking initiation, having used a smoking guide or treatment within 6 months, illustrating a desire to quit smoking, and head and neck and lung cancer sites were significant predictors of smoking cessation at 6-month follow-up Significant predictors of smoking cessation at 12-month follow-up included 15 or less cigarettes smoked per day, previously attempted a group cessation program, illustrating a desire to quit, head and neck and lung cancer sites and time since diagnosis of present cancer	None	14 patients deceased at 6-month follow -up (3.2%) and 55 (12.7%) were lost to follow-up and considered smokers for analyses At 12-month follow-up, 23 patients were deceased (5.3% and 78 (18.1%) were lost to follow-up and considered smokers for analyses	Fair/Good
Gritz et al., 1993	At 12-month follow-up, or trial completion, 70.2% (*N* = 114) of subjects (former or current smokers, enrolled in intervention or usual care) were continued abstainers (abstinent at 1-, 6-, and 12-month interviews). For those smoking at baseline, continuous abstinence was 64.6%. Of the 67 participants biochemically verified (using cotinine determination within urine), 89.6% were confirmed abstinent	There was no difference between intervention and control for number of cigarettes smoked; both groups reduced consumption by roughly half between baseline and 12-month follow-up. Although not significant, predictors of 12-month follow-up continuous abstinence include medical treatment, race, stage of change and nicotine dependence	None	At 12-month follow-up, 72 randomized subjects did not participate (38.7%). Of the original 186, 33 (17.7%) died, 4 (2.15%) progression of illness prevented participation, 16 (8.60%) dropped out, 4 (2.15%) provider not compliant with study, 1 determined not to be eligible due to literacy (0.5%), and 14 (7.53%) dropped out	Good
Schnoll et al., 2005	At 1- and 3-month follow-up, no significant differences in self-reported smoking cessation between CBT and the standard of care condition (44.9% vs. 47.3% for 1 month, 43.2% vs. 39.2% for 3 month)	In the CBT condition, patients reported greater increase in risk perceptions of smoking between baseline and one month (*p* < 0.05), and 3-month follow-up (*p* < 0.05), compared to patients in standard of care At 1-month follow-up, quit motivation was a significant predictor of smoking cessation At 3-month follow-up, cons of quitting were significant predictors of smoking cessation	None	At the 1-month follow-up evaluation, five patients were deceased (4.6% of total *N*). 28 patients (25.7%) were lost to follow-up At the 3-month follow-up, 14 patients were deceased (12.8% of total *N*). 18 patients (16.5%) were lost to follow-up	Good
Park et al., 2020	Biochemically confirmed 7-day point prevalence abstinence at 6-month follow-up was 34.5% (*n* = 51/148) for the intensive treatment group vs. 21.5% (*n* = 29/135) for the standard treatment group (difference, 13.0% [95% CI, 3.0–23.3%]; OR, 1.92 [95% CI, 1.13–3.27]; *P* < .02)	Biochemically confirmed 7-day abstinence at 3-month follow-up was 31.1% (46/148) for the intensive treatment group vs. 20.7% (*n* = 28/135) for the standard treatment group (difference, 10.3% [95% CI, 0.2–20.5%]; OR, 1.72 [95% CI, 1.00–2.96]; *P* = .048). Among those biochemically confirmed abstinent at 3 months, 29.7% (*n* = 22/74) relapsed between 3 and 6 months. Among those biochemically confirmed abstinent at 6 months, 35.0% (*n* = 28/80) quit between 3 and 6 months. Rates of continuous abstinence and 24-h quit attempts were not significantly different between treatment groups. Six-month sustained abstinence rates were 23.6% (35/148) in the intensive treatment group vs. 12.6% (17/135) in the standard treatment group (difference, 11.1% [95% CI, 2.2–19.9%]; OR, 2.15 [95% CI, 1.14–4.05]; *P* < .02)	Reported broad examination of socio-demographics as predictors of outcomes; explored socido-demographic predictors of eligibity; reported differences in the completion of the first tobacco session	82 patients did not complete the trial (27.1%)	Good
Ruebush et al., 2020	Family members were integrated into TUT for 221 (42%) patients, and 21 patients (4%) had family members present but not integrated. No significant differences were seen due to family integration based on the demographic factors. At 6 months, the 7-day point prevalence quit rate was 28% (*N* = 56/200) for patients who had family members integrated into TUT and 23% (*N* = 67/291) for patients who did not. The quit rate was not significantly higher for patients who had family members integrated into TUT (*p* = 0.105)	*There were no secondary or exploratory outcomes in this study*	None	41 patients entered a hospice or were deceased at the 6-month follow-up time point (7.7%)	Poor
Charlot et al., 2019	Overall, participants smoked fewer cigarettes after the intervention. One participant quit smoking for 14 days during the program and later relapsed. None of the participants continuously abstained from smoking. At baseline, mean weekly cigarette intake was 75.1 (SD = 52.8) cigarettes. During the last week of the intervention, mean weekly cigarette intake decreased to 50.1 (SD = 49.3) cigarettes (*p* = 0.131), and at the 3-month follow-up, there was a decrease to 44.3 cigarettes per week (*p* = 0.039)	*There were no secondary or exploratory outcomes in this study*	None	33%. 18 consented and enrolled, 1 voluntarily withdrew, 3 lost to follow-up, 2 deceased at time of follow-up	Good
Krebs et al., 2018	More participants were abstinent in the QuitIT group than in the SC arm (4/13, 30%, vs. 2/11, 18%)	*There were no secondary or exploratory outcomes in this study*	None	Experimental: 39% Control: 35%	Fair

### Representation of Black and/or Hispanic Samples

A summary of trial characteristics is noted in Table 1 and Table 3. All 10 trials included Black smokers in their sample, and enrollment rates ranged from 2–55.6%. The one study that reported high enrollment (55.6%) was a non-randomized, cohort study consisting of 18 patients [27]. Only 4/10 studies (40%) documented enrollment rates for Hispanic patients, with rates ranging from < 1–5.2% [21, 23, 25, 26].

**Table 3:** Overview of interventions in systematic review of smoking cessations interventions for Black and Hispanic cancer patients

Author (year)	Eligibility criteria	Recruitment methodology	Intervention summary	Treatment adaptations amenable to racial minorities
Browning et al., 2000	Adults with non-small cell lung cancer; daily cigarette smoker > 1 year	Patients seen in a lung cancer surgery clinic	Nurse-delivered smoking cessation intervention based on AHCPR’s smoking cessation. Strategies included setting a quit date, NRT, cessation materials, and telephone follow-up on quit date. Fac-to-face visits were conducted at follow-up	None
Nair et al., 2009	Patients treated at a hematology/oncology clinic	Patients recruited from the Central Arkansas Veterans Healthcare System Hematology/Oncology Clinic	Patients had to follow recommendations in a calendar and review the calendar with their physicians at every visit	None
Schnoll et al., 2010	Adults with a cancer diagnosis, speak English, have a telephone, and smoke at least 2 cigarettes/day on average Excluded if no current smoking; stage IV lung cancer; brain metastases; drug/alcohol dependencies; axis I psychiatric conditions; pregnant/lactating; seizure disorder; cardiac, renal, pulmonary, endocrine or neurological disorders; using an MAO inhibitor; and no longer using benzodiazepines	Proactively screened cancer patients from physician schedules	5 counseling sessions, 8 weeks of NRT and bupropion	None
Schnoll et al., 2003	19 + years old; with stage I or II of any cancer or stage III–IV lymphoma, breast, prostate, or testicular cancer; have an ECOG performance status of 0 or 1; and reported smoking cigarettes 1 + times in the past 30 days	Participating physicians at recruitment sites identified potentially eligible patients using a standard intake evaluation forms	Physician-based intervention, which includes quit advice and assistance during a clinic visit in accordance with NIH guidelines for physician-based smoking interventions	None
Gritz et al., 1993 Gritz et al., 1991	Adults newly diagnosed with squamous cell carcinomas of the head and neck; tobacco use within the past year; life expectancy of more than a year; absence of gross pathology; medical follow-up by local providers; and speak/read English	Recruited from 10 participating hospital-based medical and dental clinics in the Southern CA area	Enhanced initial advice session augmented by booster sessions integrated into first 6 monthly medical visits. Offered continued cessation support and support in high-risk relapse situations. Providers discussed subject’s receptivity of quitting	Program modeled after the “teachable moment” conceptualization
Schnoll et al., 2005	Head and neck or lung cancer diagnosis; reported smoking in past 30 days; speak English; be able to attend in-person 2-h counseling session; and be reached by phone	A member of the study team reviewed medical charts and a health educator contacted patients by phone or in the clinic	Cognitive behavioral therapy (CBT) patients were provided with an 8-week supply of the transdermal patch and a quit advice letter from their oncologist. Instructed to set a quit date at the second intervention session and received 4 counseling sessions to target psychological factors linked to tobacco cessation	None
Park et al., 2020—28	Adults who smoked 1 cigarette or more within 30 days; speak English or Spanish; phone access; and were receiving treatment for recently diagnosed breast, gastrointestinal, genitourinary, gynecological, head and neck, lung, lymphoma, or melanoma cancers	Research staff screened cancer clinic schedules from 2 NCI-designated Comprehensive Cancer	Participants were offered multiple telephone counseling sessions and their choice of 12 weeks of free FDA-approved smoking cessation medication	Intervention was offered in English and Spanish (at 1 site)
Ruebush et al.,, 2020- 32	Patients referred for Tobacco Use Treatment (TUT) by medical providers at North Carolina Cancer Hospital	Smoking patients were identified through the EHR and were referred by medical providers for TUT	At least one face-to-face session of assessment, behavioral counseling, and medication advice. Tobacco treatment specialists offered telephone follow-up. Whenever present, family members were integrated into patients’ TUT sessions	Integration of family members into treatment
Charlot et al., 2019—30	Adults with history of cancer; access to a phone; any cigarette use within the past week; and speak English. Excluded if they were actively using evidence-based tobacco cessation treatment, had a cancer prognosis less than 6 months, had untreated mental illness, or were pregnant	Boston Medical Center outpatient oncology clinic and cancer support groups. Flyers and medical assistants helped identify and recruit eligible patients	The program was conducted in eight weekly 2 h sessions of mindfulness-based group visits and tobacco treatment components. Provided additional resources for smoking cessation counseling and pharmacotherapy recommendations	None
Krebs et al., 2018—29	English-speaking; a recent cancer diagnosis or mass suspicious of cancer (within past 6 months); scheduled for surgery to remove a localized tumor; smoked cigarettes within the past 30 days; and sufficient sensory acuity and manual dexterity to use a computer game Excluded if metastatic disease; major psychiatric illness; and/or cognitive impairments	Patients who reported being current smokers were referred to the MSKCC Tobacco Treatment Program. Research staff contacted potentially eligible patients via telephone	Participants offered 4 telephone or bedside counseling sessions and in-house print cessation educational materials. Experimental condition was offered standard care (SC) in addition to having the QuitIT game installed on an iPad device. Participants were trained during hospital stay and continued 1-month post hospitalization	None

Trial designs varied widely (Tables 1 and 3). Three randomized controlled trials (RCTs) utilized physician-based smoking cessation interventions: (1) surgeon or dentist-delivered quit advice and materials [20], (2) cognitive behavioral treatment with nicotine replacement therapy (NRT) with an oncologist delivered advice letter [21], and (3) physician-delivered quit advice in accordance with NIH smoking interventions [23]. Browning and colleagues (2000) examined nurse-delivered counseling with pharmacological support and smoking cessation material; however, they used a quasi-experimental design [24]. Specifically, instead of randomizing patients to treatment arms, they compared outcomes in patients who received usual care in the 5 months preceding patients who received the nurse-delivered treatment. Three other RCTs utilized trained counselors to test the effects of behavioral counseling; however, each examined different aspects of tobacco treatment: (1) Park (2020) compared the differences between counseling intensity (short vs. long-term) [25]; (2) Schnoll (2010) tested the effects of behavioral counseling with NRT and bupropion compared to behavioral counseling with NRT and placebo [22]; and (3) Krebs (2019) compared the effects of standard tobacco treatment (i.e., 4 telecounseling sessions with educational materials) to a smoking cessation app paired with standard tobacco treatment [26]. Two trials were smaller, prospective cohort studies that (1) examined the impact of a pocket calendar and physician support [28] and (2) targeted low income and minority smokers to test the feasibility and acceptability of a mindfulness-based smoking cessation medical group visit (Charlot et al., 2019) [27]. One final study examined the feasibility of integrating families into treatment by embedding a family systems-based intervention within an existing tobacco program (Ruebush et al., 2020) [29].

### “Fit” between Trial Design and the Needs of Black and/or Hispanic Cancer Patients

Eligibility Criteria. Cancer Type: Nine of the ten studies had explicit criteria regarding cancer type and severity. Three of these focused exclusively on patients with head and/or neck cancers [20, 21] or lung cancer [21, 24] with no restrictions surrounding disease severity or health status. Four studies were open to patients with any cancer diagnosis [22, 23, 26, 29]; however, of these trials, Ruebush [29] was the only one without eligibility restrictions in cancer staging and/or ECOG status. Specifically, Schnoll [22] excluded patients with advanced cancers, those with brain metastases, and patients with chronic health conditions. Schnoll [23] limited inclusion to patients with early-stage cancer; however, it allowed patients with advanced lymphoma to participate if diagnosed with lymphoma, breast, prostrate, or testicular cancer. Eligibility was further restricted to patients who were generally healthy and functional as defined by an ECOG status ≤ 1. One study required that participants be recently diagnosed with cancer in the breast, GI, GU, GYN, head/neck, lung, lymphoma, or melanoma and currently undergoing treatment [25].

Smoking Status: Eight studies had flexible criteria for smoking status (Table 3). Four required that patients report having smoked at least one cigarette in the past 30 days [21, 23, 25, 27], and one study required that patients endorse having used tobacco in the past year [30]. A sixth study enrolled patients who smoked at least 2 daily cigarettes [22], whereas a seventh trial enrolled daily smokers, regardless of cigarette consumption, if they smoked for at least 1 year [24].

Other Criteria: Several of the studies placed additional entry restrictions based on other criteria. Four studies excluded patients based on their mental health status [22, 26, 27, 30] and five excluded patients who did not speak English [20–22, 26, 27, 30]. Four studies had specific participation requirements, including having a phone [21, 22, 25, 27] or being able to attend in-person medical care [20, 21].

Recruitment Approach*.* None of the studies described efforts to target recruitment or oversample enrollment of Black or Hispanic cancer patients (Table 3). Indeed, few offered details regarding targeted recruitment efforts. For instance, only Park (2020) described using recruitment materials in Spanish [25]. One study relied on the electronic health records (EHR) to identify participants [29], whereas another had participating physicians at recruitment sites use standard intake evaluation forms for recruitment [23]. Six studies utilized proactive recruitment efforts, including screening of physician schedules [21, 22, 25] and proactive patient outreach either in-person or by phone [20, 24, 26, 27]. Other than the location of where recruitment was taking place, one study did not identify recruitment details [28].

Recruitment Setting. Two studies did not offer information about recruitment sites [21, 22], precluding one’s ability to determine if the patients enrolled are representative of their institutions’ catchment area. The remaining eight studies were conducted in areas that had relatively large representation of Black patients and variable access to Hispanic populations [20, 23–29]. These studies were conducted in Veterans Healthcare Administration Medical Centers (VAs) [28], community-based clinics [27], Academic Medical Centers [23–25], as well as across a consortium of academic hospitals [23, 25, 26, 29]. Of these eight studies, six reported enrollment numbers that were not commensurate with the distribution of Blacks and Hispanics patients in their catchment area [23–28]. Only three studies enrolled a population of patients that was comparable to, if not surpassed, the ethnic and racial distribution in the surrounding area [20, 27, 29]. Gritz (1993) enrolled patients receiving care across a mix of academic medical centers, VAs, and county hospitals located in Southern California, an ethnically diverse region of the USA [20]. Ruebush (2020) recruited from the North Carolina Hospital Center, an academic medical center [29]. Charlot (2019) recruited from the Boston Medical Center Outpatient Clinic, part of a large, urban, safety-net hospital [27].

Treatment Adaptations*.* None of the studies directly addressed cultural/racial factors that may impact smoking behavior or outcomes among Black and/or Hispanic patients.

### Subgroup Analyses Examining Outcomes for Black and Hispanic Cancer Patients

Five studies did not report racial/ethnic differences in eligibility, enrollment, and drop-out [22, 24, 26, 28, 29]. Additionally, these studies did not examine racial/ethnic differences in cessation outcomes, although most often their sample size precluded their ability to conduct these analyses. The remaining 5 studies examined some differences in study eligibility and retention based on racial/ethnic variables. Gritz [20] investigated reasons for study ineligibility, allowing readers to consider the influence of entry criteria on Black or Hispanic patient enrollment. Similarly, they estimated racial/ethnic differences between study completers and non-completers; however, these analyses combined all racial/ethnic groups into one category. Notably, the authors looked at differences in smoking behavior by race/ethnicity only at baseline. Similarly, Schnoll et al. [23] considered the influence of race/ethnicity among other predictors on 7-day point prevalence. In a later study, Schnoll and colleagues [21] compared patients who declined participation to those who enrolled, and they found that patients who declined were more likely to be diagnosed with head/neck cancers, experience fewer physical health symptoms, reported no intention or had lower readiness to quit, and smoked fewer cigarettes. Charlot [27] noted that smokers who are part of a minority group or of a low socioeconomic status were less likely to utilize pharmacotherapy and behavioral intervention methods; however, they did not conduct formal subgroup analyses on outcomes. Park [25] analyzed differences in study eligibility, randomization and completion based on sociodemographic variables. They noted differences in session 1 completion rates (thus study randomization) between Hispanic and non-Hispanic white patients, with Hispanics being less likely to be randomized. They also acknowledged that the ability to provide pharmacotherapy free-of-cost to patients would not be available in less-resourced clinics.

## Discussion

This review is among the first to report critical gaps in the documentation and representation of two populations at high risk for experiencing poorer smoking and cancer outcomes in tobacco treatment trials. Despite our broad inclusion criteria, we identified only 10 tobacco treatment trials conducted with cancer patients that at a minimum detailed enrollment data for either Black or Hispanic cancer patients. Of these 10, only 3 studies [20, 27, 29] demonstrated accrual rates that were either proportionate to the surrounding population or highly representative of the population of Black smokers. Moreover, Hispanics remained largely under-represented across all 10 studies. These findings are disconcerting, as these groups have been evidenced to carry a disproportionate burden of disease, often presenting with more advanced illness, and exhibiting greater barriers to treatment [1, 3, 15, 16, 31]. Nonetheless, these findings corroborate existing reports documenting critical gaps in the inclusion and representation of Black and Hispanic patients in clinical trials [15, 27–31], challenging one’s ability to draw conclusions about the utility of tobacco treatments for these two cancer populations.

Several factors have been purported to explain why Black and Hispanic patients are under-represented in treatment trials, some of which include lack of trial awareness, logistical barriers (e.g., time, transportation, childcare concerns), and provider and research mistrust due to decades of discrimination, microaggressions, and mistreatment by both the medical and research community (e.g., the Tuskegee Syphillis study) [32–34, 40]. However, this review expands on these factors by highlighting how our research infrastructure (e.g., trial design and procedures) may serve as another form of structural racism, thereby widening observed disparities in trial participation and outcomes. As noted within this review, shortcomings related to how trials are designed impacts patients’ access to tobacco treatment trials. Access issues have been demonstrated to interfere with the receipt of regular medical care [35], and these challenges may extend to the clinical trial environment. Specifically, the geographical location and institutional setting of trials, often pre-determined by study funds and investigator location, may narrow the eligible pool of participants from the outset [36]. Indeed, most of the trials in this review were conducted in academic medical centers. Black and Hispanic individuals are often underinsured or uninsured, have less access to healthcare, and hold negative perceptions of academic institutions [2, 32, 37], which may deter participation.

Opening trials at community settings or in locations that are financially and logistically accessible to Black and Hispanic communities can offset these challenges and improve diversity; however, the fact that seven of the ten trials [21–28] had proportionally lower enrollment of Black and Hispanic populations than their surrounding area suggests that additional challenges may remain. In truth, none of the trials addressed these issues by either commenting on their catchment area or the representativeness of trial enrollees relative to the composition of their cancer patient population. Furthermore, none addressed concerns related to limitations in their racial/ethnic data nor discussed other logistics that may encourage trial participation in these groups (e.g., inclusion of language-appropriate materials, use of bilingual study personnel). In fact, one trial [22] did not include details regarding their geographical region.

Adequate match between the target patient population and enrollment/accrual is another feature that is central to ensuring an intervention reaches the population it intends to treat. Although some trials maintain highly selective inclusion criteria to reduce heterogeneity, this approach risks excluding populations in most need of treatment. Encouragingly, most of our eligible trials had broad inclusion criteria, which should have enhanced enrollment of Black and Hispanic smokers. In fact, all but three trials (Browning [24], Ruebush [29], Krebs [26]) used fairly flexible criteria for smoking status, which should have provided opportunities for Black and Hispanic patients, commonly light and intermittent smokers, to enroll. All trials in this review also included cancers that were most prevalent in these two populations [38]. Yet, in spite of these broad criteria, enrollment rates for these two racial/ethnic groups remained surprisingly low. It is possible that Black and Hispanic smokers were differentially screened out in six of the studies due to concerns commonly noted in these two groups, including being sicker (i.e., advanced diagnoses, additional comorbidities) [22, 23, 26, 27], speaking a language other than English [20–22, 25, 27], or endorsing psychopathology [22, 26, 27]. None of these trials examined racial/ethnic differences in trial eligibility and/or enrollment, making it difficult to ascertain if study entry criteria impeded their participation.

Additional trial-related factors that may limit the engagement of Black or Hispanic cancer patients includes the use of passive recruitment methods. Schnoll [21] relied exclusively on chart screening to identify participants, and Nair [28] omitted recruitment details altogether. Chart screening has its limitations, particularly given the evidence that race/ethnicity information is often missing or misclassified and smoking status not discussed or documented. Additionally, only Park referenced the use of non-English recruitment brochures or bilingual staff to assist with the recruitment of Spanish-speaking smokers [25].

The trials in this review also demonstrated gaps in the reporting and analysis of race. This finding is supported by Dickerson and colleagues [39] who found limited advances in the reporting of racial/ethnic analyses in cessation trials altogether. In fact, of the ten trials reviewed, only Gritz [20], Schnoll [22], and Park [25] investigated the influence of race/ethnicity among other predictors for one of their outcomes. Although low sample size and lack of sample diversity likely hindered further analyses, it is notable that only Schnoll [22] and Charlot [27] acknowledged these limitations.

Although this review is among the first to examine this question of racial/ethnic representation in smoking cessation trials for cancer patients, two groups who most at risk of are experiencing poorer smoking and cancer outcomes, several limitations are worthy of mention. Specifically, our findings are based on information drawn from 10 trials. Because we wanted to capture information that is readily available to the public, our findings reflect only information that is currently published. It is possible that other trials have been conducted with our population of interest; however, low sample sizes and publication bias (i.e., difficulty publishing null data) may have limited our access to these investigations. It is also important to note that the trials included in this review were not specifically targeting minority populations; as such, reporting of racial/ethnic variables, cultural adaptations, and related outreach efforts may have been beyond the scope of their study. Additionally, this review only included trials that took place within the USA. It is possible that trials examining these issues of race/ethnicity and smoking within cancer may have been conducted internationally.

Regardless of these limitations, given the burden of cancer and tobacco use in these two groups, there remains a need to demonstrate that tobacco treatment trials are equally effective across race and ethnicity. Efforts to address these concerns should begin at the point of tobacco trial design and extend to publication; for instance, investigators are called to design trials that account for racial/ethnic variables that may impact behavior and behavioral outcomes; expand eligibility criteria so as to maximize the inclusion of groups who would otherwise be excluded; use multimodal and culturally sensitive approaches to identify and recruit patients; engage community health centers in recruitment efforts to broaden study reach; systematically document and report reasons for trial refusal and ineligibility; and collect, report, and examine, at a preliminary level, the predictive value of racial/ethnic variables on outcomes (while acknowledging sample size limitations). In return, funding agencies and peer-reviewed journals have a shared responsibility in addressing these disparities by requiring funded trials to integrate these standards into their work prior to funding and/or publication. Lastly, the Office for Human Research Protections (OHRP) and corresponding ethics committees (IRBs) are urged to support the diversification of trial accrual by adopting policies that balance the desire to uphold patient safety and confidentiality with the need to embrace alternate, more culturally sensitive approaches that accommodate the distinctive needs of these two under-represented groups (see Table 4 for a summary of recommendations).

**Table 4:** Recommendations for future research

Unanswered questions	Researchers	Journals	Ethics committees	Funding agencies
Are we adequately enrolling Black and Hispanic cancer patients in tobacco treatment trials?	1. Expand eligibility criteria (e.g., cancer status, smoking frequency, mental health status) 2. Work collaboratively with community partners from study inception through initiation and publication 3. Include language-concordant, culturally sensitive materials 4. Design interventions that account for the influence of racial/cultural variables on smoking behavior 5. Recruit and retain bilingual and diverse staff 6. Use multiple and flexible recruitment approaches (do not limit to chart screening) 7. Document, monitor and *publish* reasons for ineligibility, refusal, study discontinuation 8. collect, report, and examine, the predictive value of racial/ethnic variables on outcomes 9. Use intervention development frameworks (like the ORBIT model) to design and conduct small studies with Black and Hispanic cancer patients to iteratively design an intervention that is accessible and leads to clinically meaningful change 10. Engage input on study design from community stakeholders	1. Accept small trials for publication (especially if they are using appropriate frameworks (like ORBIT) to design /adapt tobacco treatment interventions 2. Require the reporting of racial/ethnic sub-analyses 3. Provide room for expanding on racial/ethnic information (in an appendix or in supplemental tables) 4. Develop guidance for how to evaluate manuscripts that provide (or lack) details related to racial/ethnic minority inclusion/engagement	1. Revise existing policies to make research more accessible and appealing (e.g., simplify HIPAA language, revise the informed consent process to make it less threatening, simplify incentives without requiring social security documentation)	1. Increase funding to support the recruitment and engagement of Black and Hispanic cancer patients in tobacco treatment trials 2. Provide guidance to reviewers for evaluating proposals that include smaller trials aimed at developing/adapting tobacco treatment interventions 3. Monitor reviewer compliance with proposal review guidelines 4. Increase the funding timeline to provide additional time to recruit and engage Black and Hispanic patients in tobacco treatment trials 5. Increase funding support and training programs for early-stage investigators from diverse and disadvantaged backgrounds
Are existing tobacco treatment trials accessible to Black and Hispanic cancer patients who smoke?				
Are existing empirically supported tobacco treatments just as effective for Black and Hispanic cancer patients?				
How can we increase the inclusion of Black and Hispanic cancer patients in tobacco treatment trials?				
What adaptations are needed to enhance the acceptability tobacco treatment for these patients?				
How do we help Black and Hispanic cancer patients who smoke quit and remain quit?				

In conclusion, there are a dearth of trials that investigate the reach and effectiveness of smoking cessation treatments for Black and Hispanic patients with cancer. The results of this review suggest that existing trials do not address important gaps in the care of these populations. Given the health risk associated with continued tobacco use following a cancer diagnosis, and the relative challenges Black and Hispanic cancer patients face with tobacco and cancer treatment, there is a critical need for researchers, academic institutions, and funding agencies to increase their efforts to engage these two populations in tobacco trials.

### Supplementary Information

Below is the link to the electronic supplementary material.

Supplementary file1 (DOCX 20 KB)
