# Supplementary material for: Do Tobacco Treatment Trials Address Disparities in Smoking Outcomes Among Black and Hispanic Cancer Patients? A Systematic Review of Smoking Cessation Interventions for Black and Hispanic Patients Diagnosed with Cancer
Source: J Racial Ethn Health Disparities. 2023 Jul 19;11(4):2390–406. doi: 10.1007/s40615-023-01705-3 (PMC11236890; doi:10.1007/s40615-023-01705-3)
Supplement: Supplementary file 1 — Supplementary file1 (DOCX 20 KB) [file 40615_2023_1705_MOESM1_ESM.docx]

**Smoking Cessation Interventions Search Strategies**

**Medline Complete via EBSCO**

| S1 | (MH "Hispanic Americans+") |
| --- | --- |
| S2 | (MH "African Americans") |
| S3 | TX (Hispanic* OR Chicano* OR Chicana* OR "Spanish Speak*" OR Mexican* OR Cuban* OR "Puerto Rican*" OR Dominican* OR "Central American*" OR "South American*" OR Latino* OR Latina* OR "Hispanic American*" OR "Latin american*" OR "Mexican American*") |
| S4 | TX (Black OR Blacks OR "African descent" OR Afro-American* OR afroamerican* OR "African American*" OR "Black American*") |
| S5 | S1 OR S2 OR S3 OR S4 |
| S6 | TI (Cancer* OR Neoplasm* OR Malignan* OR Oncolog*) OR AB (Cancer* OR Neoplasm* OR Malignan* OR Oncolog*) |
| S7 | (MH "Neoplasms+") |
| S8 | S6 OR S7 |
| S9 | TI (smoking OR tobacco OR cigaret* OR nicotine) OR AB (smoking OR tobacco OR cigaret* OR nicotine) |
| S10 | TI ((cessation OR abstinence OR mediation OR intervention OR therapy OR quit OR treatment OR stop OR "quit rate*") OR AB ((cessation OR abstinence OR mediation OR intervention OR therapy OR quit OR treatment OR stop OR "quit rate*") |
| S11 | S9 AND S10 |
| S12 | (MH "Smoking Cessation") |
| S13 | (MH "Smoking/TH/DT") |
| S14 | (MH "Tobacco Use Cessation Devices+") |
| S15 | S11 OR S12 OR S13 OR S14 |
| S16 | S5 AND S8 AND S15 |

**PubMed**

((Hispanic Americans[Mesh]) OR (African Americans[Mesh]) OR (Hispanic*[Title/Abstract] OR Chicano*[Title/Abstract] OR Chicana*[Title/Abstract] OR "Spanish Speakers"[Title/Abstract] OR "Spanish Speaking"[Title/Abstract] OR Mexican*[Title/Abstract] OR Cuban*[Title/Abstract] OR "Puerto Rican*"[Title/Abstract] OR Dominican*[Title/Abstract] OR "Central American*"[Title/Abstract] OR "South American*"[Title/Abstract] OR Latino*[Title/Abstract] OR Latina*[Title/Abstract] OR "Hispanic American*"[Title/Abstract] OR "Latin american*"[Title/Abstract] OR "Mexican American*"[Title/Abstract]) OR (Black[Title/Abstract] OR Blacks[Title/Abstract] OR "African descent"[Title/Abstract] OR Afro-American*[Title/Abstract] OR afroamerican*[Title/Abstract] OR "African American*"[Title/Abstract] OR "Black American*"[Title/Abstract])) AND ((Cancer*[Title/Abstract] OR Neoplasm*[Title/Abstract] OR Malignan*[Title/Abstract] OR Oncolog*[Title/Abstract]) OR (Neoplasms[Mesh])) AND (((smoking[Title/Abstract] OR tobacco[Title/Abstract] OR cigaret*[Title/Abstract] OR nicotine[Title/Abstract]) AND (cessation[Title/Abstract] OR abstinence[Title/Abstract] OR mediation[Title/Abstract] OR intervention[Title/Abstract] OR therapy[Title/Abstract] OR quit[Title/Abstract] OR treatment[Title/Abstract] OR stop[Title/Abstract] OR "quit rate*"[Title/Abstract])) OR (Smoking Cessation[Mesh]) OR ("Smoking/drug therapy"[Mesh] OR "Smoking/therapy"[Mesh]) OR ("Tobacco Use Cessation Devices"[Mesh]))

**PsycInfo via Ovid**

| 1 | exp "latinos/latinas"/ |
| --- | --- |
| 2 | blacks/ |
| 3 | (Hispanic* or Chicano* or Chicana* or "Spanish Speakers" or "Spanish Speaking" or Mexican* or Cuban* or "Puerto Rican*" or Dominican* or "Central American*" or "South American*" or Latino* or Latina* or "Hispanic American*" or "Latin american*" or "Mexican American*").ab,ti. |
| 4 | (Black or Blacks or "African descent" or Afro-American* or afroamerican* or "African American*" or "Black American*").ab,ti. |
| 5 | 1 or 2 or 3 or 4 |
| 6 | (Cancer* or Neoplasm* or Malignan* or Oncolog*).ab,ti. |
| 7 | exp Neoplasms/ |
| 8 | 6 or 7 |
| 9 | (smoking or tobacco or cigaret* or nicotine).ab,ti. |
| 10 | (cessation or abstinence or mediation or intervention or therapy or quit or treatment or stop or "quit rate*").ab,ti. |
| 11 | 9 and 10 |
| 12 | exp smoking cessation/ |
| 13 | 11 or 12 |
| 14 | 5 and 8 and 13 |

**CINAHL Complete via EBSCO**

| S1 | (MH "Hispanics") |
| --- | --- |
| S2 | (MH "Blacks") |
| S3 | TX (Hispanic* OR Chicano* OR Chicana* OR "Spanish Speak*" OR Mexican* OR Cuban* OR "Puerto Rican*" OR Dominican* OR "Central American*" OR "South American*" OR Latino* OR Latina* OR "Hispanic American*" OR "Latin american*" OR "Mexican American*") |
| S4 | TX (Black OR Blacks OR "African descent" OR Afro-American* OR afroamerican* OR "African American*" OR "Black American*") |
| S5 | S1 OR S2 OR S3 OR S4 |
| S6 | TI (Cancer* OR Neoplasm* OR Malignan* OR Oncolog*) OR AB (Cancer* OR Neoplasm* OR Malignan* OR Oncolog*) |
| S7 | (MH "Neoplasms+") |
| S8 | S6 OR S7 |
| S9 | TI (smoking OR tobacco OR cigaret* OR nicotine) OR AB (smoking OR tobacco OR cigaret* OR nicotine) |
| S10 | TI ((cessation OR abstinence OR mediation OR intervention OR therapy OR quit OR treatment OR stop OR "quit rate*") OR AB ((cessation OR abstinence OR mediation OR intervention OR therapy OR quit OR treatment OR stop OR "quit rate*") |
| S11 | S9 AND S10 |
| S12 | (MH "Tobacco Use Cessation Products+") OR (MH "Smoking Cessation Assistance (Iowa NIC)") OR (MH "Smoking Cessation Programs") OR (MH "Smoking Cessation") |
| S13 | (MH "Smoking/TH/DT") |
| S14 | S11 OR S12 OR S13 |
| S15 | S5 AND S8 AND S14 |

**Embase.com**

| #1 | 'hispanic'/exp OR 'african american'/exp |
| --- | --- |
| #2 | hispanic*:ab,ti OR chicano*:ab,ti OR chicana*:ab,ti OR 'spanish speaking':ab,ti OR 'spanish speakers':ab,ti OR mexican*:ab,ti OR cuban*:ab,ti OR 'puerto rican':ab,ti OR 'puerto ricans':ab,ti OR dominican:ab,ti OR dominicans:ab,ti OR 'central american':ab,ti OR 'central americans':ab,ti OR 'south american':ab,ti OR 'south americans':ab,ti OR latino*:ab,ti OR latina*:ab,ti OR 'latin american':ab,ti OR 'latin americans':ab,ti |
| #3 | black:ab,ti OR blacks:ab,ti OR 'african descent':ab,ti OR 'afro american':ab,ti OR 'afro americans':ab,ti OR afroamerican*:ab,ti OR 'african american':ab,ti OR 'african americans':ab,ti |
| #4 | #1 OR #2 OR #3 |
| #5 | neoplasm'/exp |
| #6 | [deleted line] |
| #7 | cancer*:ab,ti OR neoplasm*:ab,ti OR malignan*:ab,ti OR oncolog*:ab,ti |
| #8 | #5 OR #7 |
| #9 | smoking cessation program'/exp OR 'smoking cessation'/exp OR 'nicotine replacement therapy'/exp OR 'smoking'/exp/dm_dt,dm_th |
| #10 | smoking:ab,ti OR tobacco:ab,ti OR cigaret*:ab,ti OR nicotine:ab,ti |
| #11 | cessation:ab,ti OR abstinence:ab,ti OR mediation:ab,ti OR intervention:ab,ti OR therapy:ab,ti OR quit:ab,ti OR treatment:ab,ti OR stop:ab,ti OR 'quit rate':ab,ti OR 'quit rates':ab,ti |
| #12 | #10 AND #11 |
| #13 | #9 OR #12 |
| #14 | #4 AND #8 AND #13 |
| #15 | #4 AND #8 AND #13 AND [embase]/lim |
| #16 | #4 AND #8 AND #13 AND [medline]/lim |
| #17 | #15 NOT #16 |
